# Integrated Approach for Safety Culture Factor Evaluation from a Sustainability Perspective

**DOI:** 10.3390/ijerph191911869

**Published:** 2022-09-20

**Authors:** Małgorzata Jasiulewicz-Kaczmarek, Katarzyna Antosz, Ryszard Wyczółkowski, Małgorzata Sławińska

**Affiliations:** 1 Faculty of Management Engineering, Poznan University of Technology, pl. M. Skłodowskiej-Curie 5, 60-965 Poznan, Poland; 2 Faculty of Mechanical Engineering and Aeronautics, Rzeszow University of Technology, al. Powstańców Warszawy 8, 35-959 Rzeszow, Poland; 3 Faculty of Mechanical Engineering, Silesian University of Technology, ul. Konarskiego 18a, 44-100 Gliwice, Poland

**Keywords:** safety culture, sustainability, safety culture analysis, ISM-MICMAC analysis, fuzzy TOPSIS

## Abstract

Traditionally, sustainable development has been seen as a combination of three pillars: economic, social and environmental development. In recent years, another one has been added to these three pillars, namely culture, as being indispensable in achieving sustainable development. This study proposes an integrated approach for the identification and classification of safety culture factors in the company in a sustainability context. The research design was based on the assumption that safety culture is part of organizational culture that should support the development of corporate sustainability. Firstly, the identification of the safety culture factors (SCFs) based on the literature review was presented. Then, the ISM method was used to identify the interaction between SCFs and to develop the hierarchical structure of these factors. In the next step, ISM was integrated with the MICMAC method to cluster the factors based on driving power and dependence power into four categories. Finally, safety culture factors with high driving power were rated using the fuzzy TOPSIS method from the sustainability dimension perspective. This approach was used in an automotive industry company to improve and develop the company’s practices aimed at implementing a sustainable development strategy. A sensitivity analysis was also carried out to monitor the robustness of the approach.

## 1. Introduction

The concept of corporate sustainability originates from the broader concept of sustainability. Sustainability or sustainable development became known on a global level through the report “Our Common Future”, and is defined as “development that meets the needs of the present without compromising the ability of future generations to meet their own needs” [1]. By transferring this definition to the enterprise level, sustainable development poses enterprises with a challenge that can be defined as [2]: creating value for the company, its customers and other stakeholders (economic challenge) while saving, or at least not destroying, social and ecological resources (social and environmental challenges).

Sustainable business development is considered a prerequisite for a company’s competitiveness, and companies must take measures to align their core competencies and business processes with the principles and goals of sustainable development. Meeting the challenges of sustainability requires many changes to the way companies do business. To be successful, the economic, environmental and social issues of sustainability must be integrated at all levels of the organization [3]. In recent years, companies have become increasingly aware of the challenges of sustainable development, and many manufacturing companies have adopted sustainability practices with the aim of enhancing their performance outcomes.

An important component of corporate sustainability is occupational health and safety (OHS) [4,5]. Mcquaid [6] argued that organizational practices that improve OHS conditions increase the likelihood of success in achieving SD goals. According to [7], safety and sustainable development are closely related. They both aim to achieve the same thing: resource conservation. In safety, people are a resource, while in the case of sustainable development, these resources are usually considered environmental. As in recent years more and more attention has been paid to the social dimension of sustainable development, safety is seen as an important element of corporate social responsibility and its moral obligation. More and more companies are starting to treat safety as a top priority and a key element of their strategy [8,9,10].

According to [11], actions for safety can be treated as a starting point for the operationalization of sustainable development, especially its social challenges. To respond to social challenges, organizations must undergo significant cultural changes, i.e., they must develop a sustainable-oriented organizational culture. By definition, “Culture is a fuzzy set of attitudes, beliefs, behavioral conventions, and basic assumptions and values that are shared by a group of people, and that influence each member’s behavior and each member’s interpretations of the “meaning” of other people’s behavior” [12]. According to [13], culture is considered an important factor in achieving SD, and is receiving more and more interest as the fourth dimension of SD, in addition to the environmental, social and economic dimensions. Organizational culture is considered a critical factor in achieving success in any business activity, including the implementation of the idea of sustainability. Organizational culture influences what people see, hear, feel and say. Thus, it influences the decisions and behaviors of people in the organization, and the behavior ultimately affects safety performance.

An integral part of the overall concept of organizational culture is the safety culture. In particular, the safety culture can be seen as certain aspects or parts of the organizational culture that are represented by the values, attitudes and behaviors of employees that affect the level of safety in the enterprise [14,15]. According to [16], developing a workplace safety culture has become a competitive factor for sustainable companies.

Due to the impact of safety culture on safety and environmental outcomes, the concept of safety culture has been an interesting topic for both researchers and practitioners in recent decades [17,18,19,20]. Nevertheless, there are not many studies that analyze safety culture within corporate sustainability.

This paper is novel in understanding the safety culture factors in the context of sustainability and proposes an integrated approach for the identification and classification of safety culture in the company. The aim is to analyze and rank the safety culture factors from the sustainability dimension perspective. The research question driving this study was “How can sustainability practices be improved by the development of safety culture?”, specifically “Which of the SCFs have the greatest impact on sustainability improvement in the company?”.

The framework of the study is as follows:

- Identification of the safety culture factors in a thorough literature survey.
- Developing the structural relationship framework among SCFs using the interpretive structural modelling (ISM) approach.
- Classification of the SCFs based on their driving power and dependence using MICMAC analysis.
- Ranking of the SCFs based on their importance using the fuzzy technique for order preferences by similarity of an ideal solution (F-TOPSIS) approach from the sustainability perspective.

This paper contains five sections. Section 2 explains safety culture, safety culture factors and shows how safety culture is linked to corporate sustainability. Section 3 describes the steps involved in the research methodology and also introduces ISM, MICMAC and F-TOPSIS methods. In Section 4, an application of the proposed framework is illustrated in the automotive industry. Section 5 concludes the paper. This paper is a continuation of previously undertaken work presented in [21].

## 2. Literature Review

### 2.1. Safety Culture

Safety culture is a part of organizational culture [22]. Therefore, before defining a safety culture, it is essential to understand what an organizational culture is.

Organizational culture became one of the main issues in the enterprise management system at the beginning of the 1980s [23]. The concept of “organizational culture” is interpreted differently and there is no consensus on a common definition of this term [24]. When defining organizational culture, the authors most often refer to common assumptions, norms, traditions and “beliefs which are recognized and commonly accepted as a way of life, and interpreted by the members of the organization, to constitute the way work processes are to be carried out within the organization” [25].

Many authors indicate that organizational culture impacts on customer satisfaction, productivity, communication, teamwork [26] and is the “source of sustained competitive advantage” of enterprises [27]. Moreover, organizational culture is an important environmental factor influencing whether the company’s employees want to actively participate in safety improvement activities. When analyzing the literature on safety, it is noteworthy that more and more researchers and practitioners are aware of the importance of cultural aspects of OSH management [28]. According to [29,30], the cultural context of work practices can affect safety in the same way as technology.

The term ‘‘safety culture’’ was introduced by the International Nuclear Safety Advisory Group after the Chernobyl accident to denote the management and organizational factors that are important to safety [31]. Since then, safety culture has been seen as one of the most important factors in safety performance, and is of great interest among researchers representing various scientific disciplines [32,33]

A safety culture expresses and defines the psychological and behavioral characteristics of an enterprise that can contribute to the success or failure of OSH practices [32,34,35]. In practice, the safety culture translates into safety-related activities and activities that have safety implications [36] and refers to such company management practices that are aimed at reducing unsafe behavior and positively influencing employees’ attitudes and behavior in terms of reducing risk [37,38].

The term “safety culture” does not have a universal definition (Table 1).

**Table 1:** Examples of safety culture definition.

References	Safety Culture Definitions
[39]	“the product of individual and group values, attitudes, competencies, and patterns of behaviour that determine the commitment to, and the style and proficiency of, an organisation’s health & safety programs”
[40]	“those aspects of the organisational culture which will impact on attitudes and behaviour related to increasing or decreasing risk”
[41]	“social construct used by industry and academe to describe the way that safety is being managed in organizations to avoid catastrophes and personal injuries”
[42]	“a set of organizational processes and professional practices, written rules and informal prevention, and ways of thinking, perceiving, and representing risk in organizations”
[10]	“Safety culture is a relatively stable construct consisting of collective norms, values, and assumptions that are shaped gradually over time by multilevel influences.”
[43]	“Safety culture means all material and non-material elements of a person’s well-established achievements for cultivating, recovering (when lost) and raising the level of safety of certain entities”

The importance of a safety culture as a key driver of organizational safety performance has led many industries to develop their own safety culture definitions and models. Examples of industry-specific definitions of a safety culture are presented in Table 2.

**Table 2:** Examples of industry safety culture definition.

References	Industry	Safety Culture Definitions
[44]	Nuclear	“the assembly of characteristics and attitudes in organizations and individuals that establishes that, as an overriding priority, nuclear plant safety issues receive the attention warranted by their significance”
[45]	Maritime	“culture in which there is considerable informed endeavour to reduce risks to the individual, ships and the marine environment to a level that is as low as is reasonably practicable”
[46]	Railway	“refers to the interaction between the requirements of the Safety Management System (SMS), how people make sense of them, based on their attitudes, values and beliefs, and what they actually do, as seen in decisions and behaviours”

The review of the definition of a safety culture presented above shows that regardless of the differences resulting from the interdisciplinary nature of this concept, common to all definitions is the fact that each of them emphasizes that safety culture is a proactive approach to safety. This proactive approach to safety can support the company to better understand existing problems and their future solutions and build adequate capacity to implement initiatives to improve safety, improve the quality of working life and manage occupational health and safety (OSH) more effectively.

OSH and thus the safety culture was disclosed as the most important in terms of sustainability [47]. In the literature and in practice, the most frequently cited interpretation of sustainability is the triple bottom line (TBL) [48]. TBL has three pillars: economic sustainability, aimed at improving financial results (securing liquidity and ensuring profit); environmental sustainability, which relates to the protection of the environment and rational management of natural resources; and social sustainability, which contributes to the development of human and social capital [49]. Despite the fact that the TBL approach is very popular, more and more often in the approach to sustainability, the authors pay attention to issues related to culture, pointing out that taking into account culture as the fourth dimension is necessary to achieve sustainable development [13]. According to [50], culture plays the role of a mediator between the environmental, social and economic pillars of sustainability. Moreover, by considering this aspect of sustainability, we can better understand and learn about the internal characteristics of the company, such as management commitment, approach to innovation, environmental issues and safety.

From the point of view of social sustainability, a strong safety culture improves safety performance by reducing the severity and frequency of occupational incidents, reducing the number of accidents and near misses, and increasing the effectiveness of the company’s safety improvement programs, such as behavior-based safety (BBS) [51,52,53,54,55].

From the point of view of environmental sustainability, Ref. [56] argues that “organizations with a positive safety culture are more likely to adopt an environmental sustainability perspective, implement environmentally friendly practices, and improve their environmental performance”. Accidents at work are sometimes related to environmental problems. Therefore, reducing/eliminating hazards and risks in the workplace can support companies in implementing good environmental practices, and thus prevent the generation of waste and pollution, which in turn will improve their environmental performance.

Safety programs and culture are important not only from a health and environmental point of view, but are also crucial to sustainable supply chains, high-quality production and organizational productivity (economic sustainability) [57]. According to the results of the analysis carried out by [58], the increase in the number of accidents at work reduces sales per employee, operating profit per employee, the ratio of operating profit to sales and the rate of sales growth by a statistically significant level.

Many companies around the world are beginning to take an interest in the concept of a safety culture [34]. The basic question that managers must answer in order to improve the safety culture in their company is: what factors influence the safety culture?

### 2.2. Safety Culture Factors

Scientists around the world have identified numerous factors of safety culture as factors for the success of safety management programs [20]. For instance, Ref. [59] identified four groups of safety culture factors in the railway industry and for each of them defined safety culture dimensions: (1) environmental factors (dimension: safety environment; safety rule); (2) organizational factors (dimensions: safety commitment; safety training; safety system; safety leadership; health activities; risk management; safety encouragement and punishment; performance measurement; contractor management; management of change; procurement management; safety communication); (3) personal factor (dimensions: safety knowledge; worker participation); and (4) psychological factors (dimensions: safety awareness and attitude; safe behavior). According to [60], the development of a safety culture depends on management commitment, open and honest communication, employee participation, health and safety education, accident reporting and the analysis of incidents and potential incidents, the motivation and recognition of employees who operate safely, cooperation between employees, trust between management and employees and between employees of various departments and organizational levels. In [61], the authors conducted a systematic review and examined the factors influencing the psychological, situational and behavioral dimensions of safety culture in mining. A review of proposals in the literature of factors influencing the safety culture is presented in Table 3.

Several factors have been identified as supporting the development of a positive safety culture within various industries. Based on the review (Table 3), the following safety culture factors can be considered as key: management commitment, communication, training and education on OHS, reporting system/analysis of accidents.

The commitment of the management is an expression of personal interest and concern for the safety of employees, compliance with health and safety regulations and treating safety issues on an equal footing with other tasks performed by individual organizational units. Open and honest communication is based on communicating with others, persuading, learning, listening, speaking, and reaching a compromise or consensus. Communication concerns all employees at all levels of the organizational structure. At the same time, the quality of communication in a company has a direct impact on employee motivation, sense of belonging, job satisfaction as well as efficiency and effectiveness. In addition, it is much easier to get feedback on what has been done well and what can still be improved [10]. In such organizations, reporting errors and near misses becomes an important part of everyday work and promotes a better understanding of security issues. It is also conducive to building training plans adequate to the needs of the company, increasing safety awareness among employees. Given the context in which companies operate today, namely intense competition, more demanding consumers, pressure from stakeholders, resource scarcity and technological advances, the adoption of sustainable practices is imperative. According to [67], “Corporate sustainability needs to be considered strategic and, thus, must be embedded in the culture and values of the organisation”. Because of the close relationship between safety culture and organizational culture [61], the development and improvement of the safety culture affect the implementation of and the challenges relating to sustainable development [62].

Therefore, it is necessary to understand the relationship between the factors influencing the safety culture and determine their importance from the perspective of the dimensions of sustainable development.

## 3. Research Methodology

The objective of this study was to explore the interrelationship existing between factors that impact the safety culture and rank these factors from a sustainability perspective. For these purposes, we chose a combined ISM-MICMAC and F-TOPIS framework for providing a hierarchically structured way of identified drivers as well as finding the inner relationship between them.

The research methodology consisted of three steps. The first step was to identify the factors influencing the safety culture in the enterprise. Then, in the second step, the ISM-MICMAC analysis was used. The ISM approach was applied to evaluate the relationship between the identified safety culture factors. After that, the MICMAC approach was applied to elaborate the relationship between the factors based on their influence and dependence value.

As the ISM-MICMAC approach did not rank the factors, the F-TOPSIS method was used in the next step. An F-TOPSIS approach was used to rank the most important safety culture factors that have an impact on corporate sustainability. The integration of the F-TOPSIS method with ISM-MICMAC will enable a better understanding of safety culture factors from a sustainability perspective. The descriptions of the methods used in this research are presented below.

### 3.1. ISM-MICMAC Analysis

Interpretive structural modelling (ISM) was developed by Warfield in 1973 to analyze complex socioeconomic systems [68]. When considering a complex system, many factors may be related to the system or problem in question. The ISM process consists of decomposing a complex system into several components using practical experience and expert knowledge. The result of this decomposition is a structured model or graphical representation of the system. This helps us to understand the problem better, as the direct and indirect relationships between factors describe the situation much more accurately than the individual factor taken into account.

As the complexity of the structure of the analyzed systems/problems increases, the ISM method is gaining popularity. In recent years, the ISM method has been applied in various research fields [69]. Ai et al. [70] employed ISM to build a hierarchical structure of factors influencing work safety. Liu at al. [71] applied ISM to ascertain the interrelations among critical success factors for safety management in subway construction. Wang et al. [72] used a hybrid process combining Rasmussen’s AcciMap approach and ISM to determine the potential contributory factors for industrial accidents. Xu and Shi [73] used ISM to analyze factors affecting SMEs employees’ safety production behavior. Wang et al. [74] applied ISM and MICMAC methods to delineate the hierarchy of factors influencing the unsafe behaviors of miners and to determine the interdependence between these factors.

The steps of ISM development are presented below:

**Step 1**: Identify the variables/factors which are relevant to the system/problem under study

**Step 2**: Create a structural self-interaction matrix (SSIM) to explain pairwise relationships between the factors studied.

**Step 3**: Create a reachability matrix based on SSIM and test transitivity. Transitivity is an underlying assumption in ISM that states that if A is related to B and B is related to C, then A is necessarily related to C.

**Step 4**: Partition the reachability matrix into different levels.

**Step 5**: Convert the reachability matrix into conical form by clustering factors at the same level across the rows and columns of the final reachability matrix.

**Step 6**: Design the graph and remove transitive links based on the relationships explained in the reachability.

**Step 7**: Convert the resultant digraph into an ISM-based model by replacing element nodes with the statements.

The result of the above procedure is a structured hierarchical model/graphical representation of the problem/system under consideration, which enables a better understanding of the problem and more effective communication with others.

An important supplement and extension of the ISM method is the MICMAC (Matrice d’Impacts Croisés Multiplication Appliquée á un Classement) analysis [75]. The purpose of the MICMAC analysis is to investigate the driving force and the strength of the relationship between the factors describing the problem under study.

MICMAC analysis works on the principle of the multiplication properties of matrices (if X directly affects Y and Y directly affects Z, then any change that affects X can affect Z) [76]. Based on this principle, the MICMAC method examines the impact and relationships between factors and classifies them into independent, related, dependent and autonomous clusters.

MICMAC method is divided into the following steps [77]:

**Step 1**: Identify the variables/factors.

**Step 2**: Construct a structural analysis matrix. Matrix cells define the relationship/influence between the analyzed variables. The impact can be defined as follows: 0 as no influence between variables; 1 as a weak influence between variables; 2 as a strong influence between variables; 3 as a very strong influence between variables; and P as the potential influence between variables.

**Step 3**: Direct impact analysis. In direct analysis, the total direct influence and dependence of a variable in the system are assessed directly from the direct and indirect matrix.

**Step 4**: Indirect impact analysis. In indirect analysis, the classification increases the power of the matrix by means of multiple multiplications. The links and feedback loops connecting the factor units are used to analyze the spread of interactions in the system. The result of the analysis is the setting of priorities, which allows for the identification of hidden influences that are difficult to determine by experts in direct assessment.

**Step 5**: Assign the variables to the four clusters based on their influence and dependence power [78]: cluster I—“Autonomous factors”—factors with weak influence and weak dependence power; cluster II—“Dependence factors”—factors with strong influence and weak dependence; cluster III—“Linkage factors”—factors with strong influence and dependence and cluster IV—“Independent/Driving factors”—factors with strong influence, but weak dependence.

In the next step, the results of ISM-MICMAC analyses are used as an input to F-TOPIS method.

### 3.2. Fuzzy Technique for Order Preference by Similarity to the Ideal Solution (F-TOPSIS)

The Technique for Order Preference by Similarity to Ideal Solution (TOPSIS) method is a useful and straightforward method to choose the best option based on calculating the distance from the positive and negative ideal solution. According to [79,80], TOPSIS is one of the most widely used and popular multi-criteria decision-making (MCDM) methods. In the classical approach, TOPSIS relies on assessments provided by the decision maker in the form of precise numerical values. However, in the case of many complex, real decision-making problems, the data provided by a group of decision-makers (experts) is imprecise (usually linguistic) [80]. In such a situation, it is important to capture the uncertainty of expressed opinions and preferences.

Considering the fuzziness in the decision data and group decision-making process, [81] proposed using linguistic variables to evaluate the weights of all criteria and to evaluate each alternative against each criterion. Each decision-maker evaluates the criteria/rank of factors by selecting the appropriate “words” from the language scale. The resulting linguistic scores of the criteria are then converted into fuzzy values. For this purpose, it is necessary to build an appropriate membership function, representing the adopted scale of linguistic assessments. In practice, triangular fuzzy numbers (TFN) are most often employed [82,83].

A triangular fuzzy number $\tilde{A}$ is denoted by a triplet $\left(l,\;m,\;u\right)$ and is defined by a membership function ${µ}_{\tilde{A}}\left(x\right):R\to \left[0,\;1\right]$ as follows:

(1) $${µ}_{\tilde{A}}\left(x\right)=\left\{\begin{array}{c} 0\;\;\;\;\;\;\;\;\;\;\;\;\;\;\;\;\;\;\;\;\;\;\;\;\;x<l\\ x-l/m-l,\;l\leq x\leq \;m\;\\ x-u/m-u,\;m\leq x\leq u\;\\ 0\;\;\;\;\;\;\;\;\;\;\;\;\;\;\;\;\;\;\;\;\;\;\;\;\;\;\;x>u \end{array}\right.$$

with l≤m≤u, where *l*, *m*, and *u* are real numbers representing the lower, medium, and upper limits, respectively.

The algebraic operators of two TFNs $\tilde{A}=\left(l_{1},\;m_{1},\;u_{1}\right)$ and $\tilde{B}=\left(l_{2},\;m_{2},\;u_{2}\right)$ are expressed as follows:

(2) $$\tilde{A}⊕\tilde{B}\approx \left(l_{1}+l_{2},\;m_{1}+m_{2},\;u_{1}+u_{2}\right),\;$$

(3) $$\tilde{A}⊖\tilde{B}\approx \left(l_{1}-u_{2},\;m_{1}-m_{2},\;u_{1}-l_{2}\right),\;$$

(4) $$\tilde{A}⊗\tilde{B}\approx \left(l_{1}l_{2},\;m_{1}m_{2},\;u_{1}u_{2}\right),\;$$

(5) $$\tilde{A}⊘\tilde{B}\approx \left(l_{1}/u_{2},m_{1}/m_{2},u_{1}/l_{2}\right)$$

(6) $$\tilde{A^{-1}}\approx \left(1/u_{1},\;1/m_{1},1/l_{1}\right)\;\mathrm{for}l,\;m,\;u>0$$

(7) $$k⊗\tilde{A}\approx \left(kl_{1},\;km_{1},\;ku_{1}\right),\;k>0,\;kR,$$

The steps of the F-TOPSIS method used in this study are similar to the approach in [81], and can be presented in the following way:

**Step 1:** Identify the relevant criteria.

**Step 2:** Choose linguistic terms for the importance weight of the criteria and the linguistic ratings for alternatives with respect to the criteria (Table 4 and Table 5) [81].

**Step 3:** Aggregate the weight of criteria to obtain the aggregated fuzzy weight ${\tilde{w}}_{j}$ of criterion *Cj* and collect the opinions of decision-makers to obtain the aggregated fuzzy rating ${\tilde{x}}_{ij}$ of alternative *A_i_* under criterion *Cj*. If a decision group has *K* persons, then the importance of the criteria and the rating of alternatives with respect to each criterion can be calculated as:

(8) $${\tilde{w}}_{j}=\frac{1}{K}\left[{\tilde{w}}_{j}^{1}\left(+\right){\tilde{w}}_{j}^{2}\left(+\right)\cdots \left(+\right){\tilde{w}}_{j}^{K}\right],$$

(9) $${\tilde{x}}_{ij}=\frac{1}{K}\left[{\tilde{x}}_{ij}^{1}\left(+\right){\tilde{x}}_{ij}^{2}\left(+\right)\cdots \left(+\right){\tilde{x}}_{ij}^{K}\right],$$

where ${\tilde{w}}_{j}^{K}$ and ${\tilde{x}}_{ij}$ are the rating and the importance weight of the *K*th decision maker, respectively.

**Step 4:** Construct the fuzzy decision matrix.

(10) $$\tilde{D}={\left[{\tilde{x}}_{ij}\right]}_{m\times n},\;\tilde{W}=\left[{\tilde{w}}_{1},\;{\tilde{w}}_{2},\;\ldots ,\;{\tilde{w}}_{n}\right],\;i=1,\;2,\ldots ,\;m,\;j=1,\;2,\;\ldots ,\;n,$$

where ${\tilde{x}}_{ij},\;\forall i,\mathrm{and}\;j$ are linguistic variables, ${\tilde{x}}_{ij}=\left(a_{ij},\;b_{ij},\;c_{ij}\right)$ and ${\tilde{w}}_{j}=\left(w_{j1},\;w_{j2},\;w_{j3}\right)$

**Step 5:** Normalize the fuzzy decision matrix.

(11) $$\tilde{R}={\left[{\tilde{r}}_{ij}\right]}_{m\times n},\;i=1,\;2,\ldots ,\;m,\;j=1,\;2,\;\ldots ,\;n,$$

where

(12) $${\tilde{R}}_{\mathrm{ij}}=\left(\frac{a_{\mathrm{ij}}}{c_{j}^{*}},\frac{b_{\mathrm{ij}}}{c_{j}^{*}},\frac{c_{\mathrm{ij}}}{c_{j}^{*}}\right),c_{j}^{*}=\underset{i}{\max}\;c_{\mathrm{ij}}\;j\in B,\;(\mathrm{benefit}\;\mathrm{criteria})$$

(13) $${\tilde{R}}_{\mathrm{ij}}=\left(\frac{a_{j}^{-}}{c_{\mathrm{ij}}},\frac{a_{j}^{-}}{b_{\mathrm{ij}}},\frac{a_{j}^{-}}{a_{\mathrm{ij}}}\right),a_{j}^{-}=\underset{i}{\min}\;a_{\mathrm{ij}}\;j\in C,\;(\mathrm{cost}\;\mathrm{criteria})$$

**Step 6:** Construct the weighted normalized fuzzy decision

(14) $$\tilde{V}={\left[{\tilde{v}}_{ij}\right]}_{m\times n},\;i=1,\;2,\ldots ,\;m,\;j=1,\;2,\;\ldots ,\;n,$$

where ${\tilde{v}}_{ij}={\tilde{r}}_{ij}\left(\cdot \right){\tilde{w}}_{j}.$

**Step 7:** Compute the fuzzy positive ideal solution (FPIS) and fuzzy negative ideal solution (FNIS)

(15) $$A^{*}=\left({\tilde{v}}_{1}^{*},\;{\tilde{v}}_{2}^{*},\;\ldots ,\;{\tilde{v}}_{n}^{*}\right),$$

(16) $$A^{-}=\left({\tilde{v}}_{1}^{-},\;{\tilde{v}}_{2}^{-},\;\ldots ,\;{\tilde{v}}_{n}^{-}\right),$$

where ${\tilde{v}}_{j}^{*}=\left(1,\;1,\;1\right)$ and ${\tilde{v}}_{j}^{-}=\left(0,\;0,\;0\right),\;j=1,\;2,\;\ldots ,n.$

**Step 8:** Calculate the distance of each alternative from FPIS and FNIS.

(17) $$d_{i}^{*}=\sum_{j=1}^{n}d({\tilde{v}}_{ij},{\tilde{v}}_{j}^{*}),\;i=1,2,\;\ldots ,\;m,$$

(18) $$d_{i}^{-}=\sum_{j=1}^{n}d({\tilde{v}}_{ij},{\tilde{v}}_{j}^{-}),\;i=1,2,\;\ldots ,\;m,$$

where *d* (۰,۰) is the distance measurement between two fuzzy numbers.

**Step 9:** Compute the closeness coefficient ($CC_{i}$) of each alternative as follows:

(19) $$CC_{i}=\frac{d_{i}^{-}}{d_{i}^{*}+d_{i}^{-}},\;i=1,2,\;\ldots ,\;m,$$

**Step 10:** Rank the alternatives. The best alternative is closest to the FPIS and farthest from the FNIS

## 4. Results and Analysis

**Figure 1:**
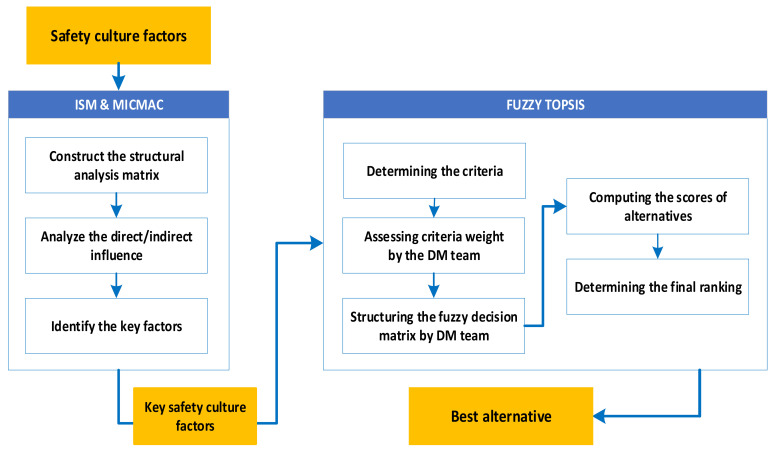
The model of the research.

The proposed framework was performed according to the methodology presented in Figure 1 in a manufacturing company from the automotive industry.

The selection of a company from this industry was driven by two factors: first, the scope of the industry’s impact, and second, the requirements for processes and products in this industry. The scope of the industry’s impact is related to its impact on other industries and society, while the requirements set for processes and products refer to standards of their implementation determined by customers and legal regulations. The automotive industry is a major stakeholder in several areas of human life, and because of their environmental and social risk (Table 6), they play a significant role in sustainability [84].

The automotive sector is considered one of the most important in a country’s economy. In terms of the value of production sold, the automotive industry ranks second after the food industry, ahead of other segments of the manufacturing industry. The strength of the automotive sector leverages other sectors of the economy (e.g., steel industry, aluminum sector, plastics). The automotive industry is responsible for the development of technological innovation and management, and for the huge change in industrial production processes. Technological changes and the growing popularity of strategies focused on so-called “sustainable development” are, according to [86], the main factors that revolutionize global automotive markets. Environmental legislation affects both the design and performance of automotive industry products and the ways in which they are manufactured [87]. Nunes and Bennett [86] investigated green operations initiatives in the automotive industry that were documented in the environmental reports of selected companies. They found that car manufacturers are pursuing a wide range of green operations practices such as eco-design, green supply chains, green manufacturing, reverse logistics, and innovation. The environment is not the only sector at which the automotive industry has aimed its actions. Continued upskilling of its workforce enables the industry to stay competitive and prepare for future challenges. For example, as reported by The Society of Motor Manufacturers and Trades [88], the number of formal training days per employee in the UK automotive industry increased in 2017 by 23.3%, from 3 to 3.7 days. The safety of staff is of paramount importance to the industry; hence, a large amount of training is dedicated to job induction and accident prevention [89].

Automotive companies are focused on quality (e.g., ISO 9001, IATF), the environment (ISO 14001), and workplace safety (e.g., ISO 45001), and apply sustainable practices intensively in their internal operations and the supply chain in order to reduce the impact generated by production and distribution processes [88,89,90]. Therefore, the issues relating to human capital, quality of work life, occupational health and safety and safety culture cannot be avoided [42,66,91,92,93,94].

To evaluate the importance of identified safety culture factors, the research in the automotive SME was conducted. The main requirement of the company’s clients (beyond quality of the products) is compliance with the sustainability policy and objectives and the Code of Conduct.

The sustainability goals established by the company’s management are aimed at achieving the economical use of resources, reducing energy consumption, and preserving employees’ safety and wellbeing. Therefore, the improvement activities are taken in the company to identify the most important factors for assumed sustainability goal achievement.

### 4.1. Identification Factors Affecting Safety Culture

The modelling and improvement of safety culture is not an easy task, and evolution from a reactive culture to a proactive one is even more difficult. Manufacturing companies face various factors during these processes. So, for the successful improvement of safety culture and to support sustainability performance in any company, there is a need to identify and analyze factors which affect the safety culture in a company. The criteria adopted for the evaluation that may affect the safety culture often differ from one organization to another. They are often dependent on the type of assets (resources) as well as the adopted rules of the organization.

In this research, the identification of safety culture factors was completed by the authors (based on the literature) in consultation with a team from the company based on corporate sustainability policy and objectives on the one hand, and literature analysis and the authors’ experience on the other (Table 7).

These ten factors (F_1_ to F_10_) outlined above will be further analyzed.

### 4.2. ISM-MICMAC Analysis

This section includes the two following steps:

(1) ISM analysis, and
(2) MICMAC analysis.

ISM analysis was performed for ten safety culture factors (see Table 6). The contextual relationship among the factors was developed through brainstorming by four experts (three from the industry: occupational health and safety specialist, environmental management specialist, Kaizen Leader and one from academia).

The experts were asked to assess the degree of influence between each pair of factors. Four symbols were used to denote the direction of the relationship between the two factors (*i* and *j*): V: factor *i* influences factor *j*; A: factor *i* is influenced by factor *j*; X: factor *i* and *j* influence each other; and O: factor *i* and *j* do not influence each other, since they are unrelated. Using the team’s expertise, the SSIM was developed (Table 8).

Examples of each category of relationship are given below:

(1) Factor F_1_ (Management commitment) would augment factor F_3_ (Rewards and Recognition). When there is a lack of commitment from top management towards safety culture, the money required to implement such programmes will be hard to come by and thus the relationship is V.
(2) Factor F_2_ (Communication) is augmented by factor F_8_ (Attitudes towards OHS regulations, so the relationship is A.
(3) Factor F_2_ (Communication) and factor F_4_ (Trust between managers and employees) augment each other, so the relationship is X.
(4) No direct relationship appears to exist between factor F_8_ (Attitudes towards OHS regulations) and factor F_10_ (Monitoring employees behavior), so the relationship is O.

The SSIM is converted into a binary matrix (called the initial reachability matrix (Table 9) by substituting V, A, X and O with 1 and 0 according to the following rules:

If the (*i, j*) entry in the SSIM is:

- V, then the (*i, j*) entry in the reachability matrix becomes 1 and the (*j, i*) entry becomes 0.
- A, then the (*i, j*) entry in the reachability matrix becomes 0 and the (*j, i*) entry becomes 1.
- X, then the (*i, j*) entry in the reachability matrix becomes 1 and the (*j, i*) entry also becomes 1.
- O, then the (*i, j*) entry in the reachability matrix becomes 0 and the (*j, i*) entry also becomes 0.

**Table 9:** Initial reachability matrix.

	F_1_	F_2_	F_3_	F_4_	F_5_	F_6_	F_7_	F_8_	F_9_	F_10_
**F_1_**	1	1	1	1	1	1	1	1	1	1
**F_2_**	0	1	1	1	1	1	1	0	0	1
**F_3_**	0	0	1	1	0	1	1	1	0	0
**F_4_**	0	1	0	1	1	1	1	1	1	0
**F_5_**	0	1	0	0	1	0	1	0	0	0
**F_6_**	0	1	1	0	0	1	1	1	1	1
**F_7_**	0	0	0	1	1	0	1	1	0	1
**F_8_**	0	0	1	1	0	0	0	1	0	0
**F_9_**	0	1	0	1	0	0	0	0	1	1
**F_10_**	0	1	0	0	0	0	0	1	0	1

To obtain the final reachability matrix (FRM), the concept of transitivity was introduced (Table 10).

After the FRM was composed, reachable and antecedent sets were defined and then their intersections were obtained (Table 11).

**Figure 2:**
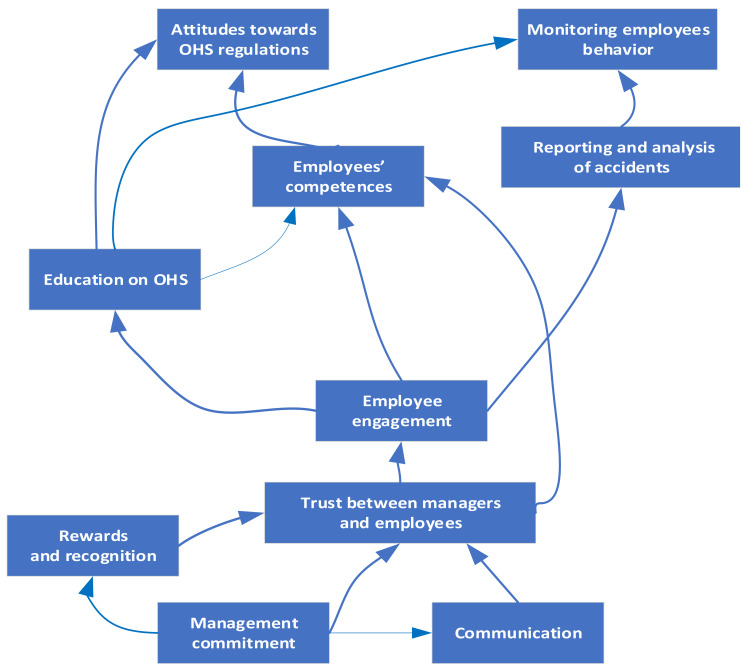
ISM model of the safety culture factors.

Then, the ISM was generated by means of vertices or nodes and lines of edges from the FRM (Table 9). If there is a relationship between factors *i* and *j*, this is shown by an arrow which points from *i* to *j*. This graph is called a directed graph or digraph. After removing the transmittivities, the digraph was finally converted into ISM, as shown in Figure 2.

The model presented in Figure 2 shows the interrelationships between various SCFs and defines the hierarchy between them. Thanks to this, it is possible to identify the main factors that should be taken into account in the first place in order to efficiently develop and improve the safety culture in the company. Regardless of the number of identified levels resulting from the ISM analysis, on the Figure 2 three areas of building a safety culture in a company can be defined. The first area includes four factors (F_1_, F_2_, F_3_, F_4_), which are prerequisites for building a safety culture. The result of the undertaken actions is building people’s engagement (the second area—factor F_5_). Engagement is considered as an important strategic tool to attract, motivate and retain the employees to achieve the success of the improvement projects. As a consequence, the engagement of the employees will have an impact for the proper perception and understanding of the importance of factors in the third area (F_6_, F_7_, F_8_, F_9_, F_10_).

In Table 9 of the FRM, the driving power (the total number of factors—including itself—that can be influenced by it) and the dependence of the relationship (the total number of factors—including itself—that can affect it) are shown for each factor. These driving powers and dependencies will be used in the MICMAC analysis.

The MICMAC system is a specialized computer program developed by M. Godet [124], serving as a tool for structural analysis. The use of the MICMAC program allows the analysis of complex systems with many driving forces, enabling the transition from a comprehensive mapping of the system to its simplified form. The major highlight of the ISM-MICMAC approach is that it helps to analyze the problem situation through graphical representation and a structured model [125].

**Figure 3:**
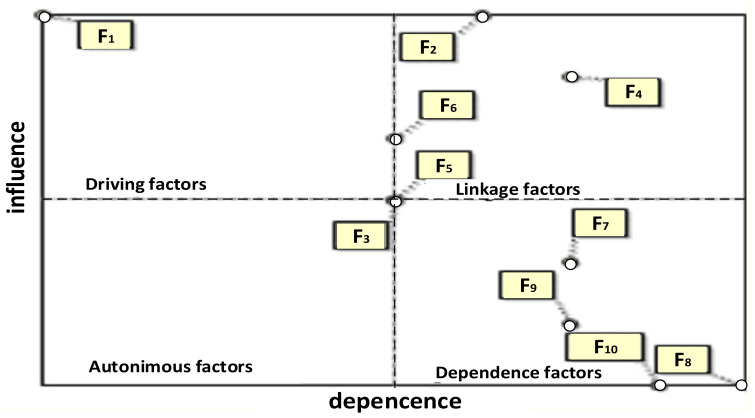
Direct influence map according for the safety culture factors with identified impact.

In MICMAC analysis, the SCFs are divided into four groups (Figure 3).

The first group consists of autonomous factors (weak driving power and weak dependence power), separated from the system. As shown in Figure 3, none of the factors belong to this group. To the second group “dependence factors” (weak driving power and high dependence power) belong the factors F_7_, F_8_, F_9_ and F_10_. They are particularly sensitive to changes in driving and linkage factors. To the third group “linkage factors” belong F_2_, F_4_ and F_6_. These factors combine a high influence power and a high degree of dependence power. Factor F_1_ belongs to the “driving factors”. This factor has a very strong influence power on the system and is difficult to control. Additionally, there are also regulatory factors on the map (F_3_ and F_5_). These factors are characterized by a small influence power on the system, but they can be important in achieving strategic goals in the enterprise.

Due to the lack of unambiguous identification of the SCF factors, in the next step, the power of the impact for individual factors was determined, and then the direct and indirect influences were analyzed.

The power of the influence of safety culture factors was assessed on a three-point scale, where: “0”—no impact; “1”—weak influence; “2”—medium impact (significant, but not decisive); and “3”—large (decisive) impact). In every case, representatives were asked if the changing of the first factor (listed in a row) would cause a direct change to the second factor (listed in a column). Each factor was assessed with respect to the rest of the factors (Table 12).

**Figure 4:**
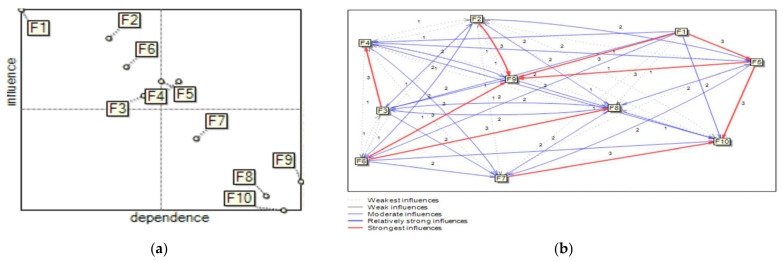
Direct influence/dependence map (**a**) and graph (**b**) for SCFs with a defined power of impact.

Based on $DI_{i}$ and $DP_{i}$, 10 anlyzed factors supporting the development of sustainability using MICMAC were classified into four clusters. Figure 4 presents the direct influence map and graph for the analyzed SCFs.

When analyzing the results presented on the map in Figure 4, it should be noted that considering the power of the impact, some factors changed their membership to particular groups. The difference is most noticeable in the third and fourth groups, in the “linkage factors” and “driving factors”. Currently, in the fourth group of “driving factors”, apart from the factor F_1_, there are also the factors F_2_, F_3_ and F_6_. These SCFs have weak dependence, but strong driving power. However, in the third group of “linkage factors”, there is only the factor F_4_. Moreover, the factor F_5_ is located near the center line of the map, and consequently is part of the “linkage factors” group or the “driving factors” group.

The second group, the “dependence factors”, still consists of the factors F_7_, F_8_, F_9_ and F_10_ as the dependent SCFs that have weak driving power but strong dependence. These factors still have a weak influence but are strongly dependent on the others. Their strong dependence dictates that they require all of the other SCFs to minimize the effect of these factors during safety culture improvement. The management should therefore assign high priority to these SCFs. Still none of the factors belongs to the fourth group.

Figure 4 shows that other factors strongly depend on factors F_9_ and F_10_. Moreover, F_9_ depends the most on the factors F_2,_ F_5_ and F_6_. The factor F_10_ depends the most on the factors F_5_ and F_7_.

In the further analyses, indirect impacts were taken into consideration. Based on the matrix of direct influence, the matrix of indirect influence was determined (Table 13). After four iterations, I = 4, stability of the indirect matrix was obtained for the analyzed factors. The analysis of indirect impacts made it possible to additionally take into account the hidden impacts.

**Figure 5:**
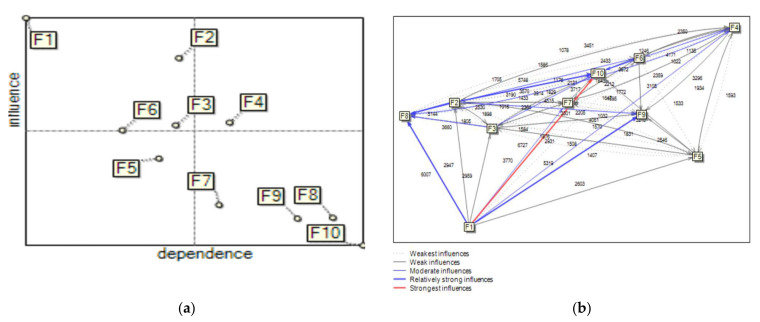
Indirect influence map (**a**) and graph (**b**) for SCFs with a defined power of impact.

The high values of the elements of the indirect influence matrix indicate an increase in the strength of the interaction between the analyzed factors. Figure 5 presents the indirect influence map and graph for SCFs with a defined power of impact.

Analyzing the indirect impact map, it can be seen that the most noticeable difference is the change in the position of factor F_5_. Currently, that factor belongs to the first group, “autonomous factors”. This factor has a weak driving power and very weak dependence, and this factor is relatively separate from the system. In previous analyses (Figure 3 and Figure 4), none of the identified factors belonged to this group, which meant that none of them were separate from the other factors. Therefore, management had to pay attention to all identified factors.

**Figure 6:**
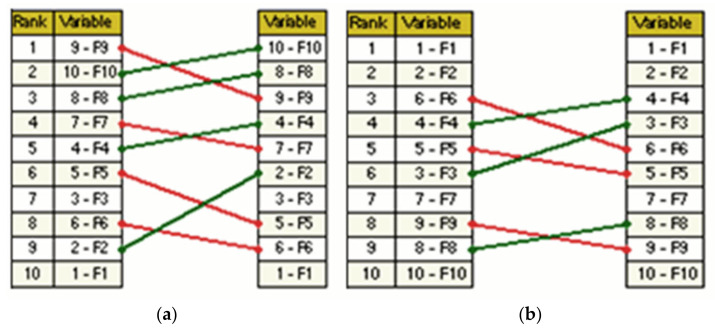
The ranking comparison of SCFs among factors according to their influences (**a**) and dependencies (**b**).

In Figure 6, the ranking comparison among factors according to their direct and indirect influences and dependencies is presented. It can observed that there is some displacement among factors once in the indirect influences. Furthermore, for the four factors F_9_, F_7_, F_5_ and F_6_, the value of the impact decreased. At the same time, for another four factors (F_10_, F_8_, F_4_ and F_2_), the value of the impact increased. The greatest displacement is noticeable for the factor F_2_. In the matrix of direct influence, this factor was in ninth place, while here it moved to sixth place. Meanwhile, two factors, F_1_ and F_3_, did not change their position.

As shown in Figure 6, four factors (F_1_, F_2_, F_7_ and F_10_) did not change their position. The factors F_4_, F_3_ and F_8_ increased their position quite significantly in the indirect dependence matrix. The greatest displacement is noticeable for the factor F_3_. This indicates that this factor (Rewards and Recognition) is the priority at this time. Moreover, three factors, F_5_, F_6_ and F_9_, decreased their position quite significantly in the indirect dependence matrix. This indicates that these factors (F_5_—Employee engagement, F_6_—Education on OHS and F_9_—Analysis of accidents) are not the priority at this time.

**Figure 7:**
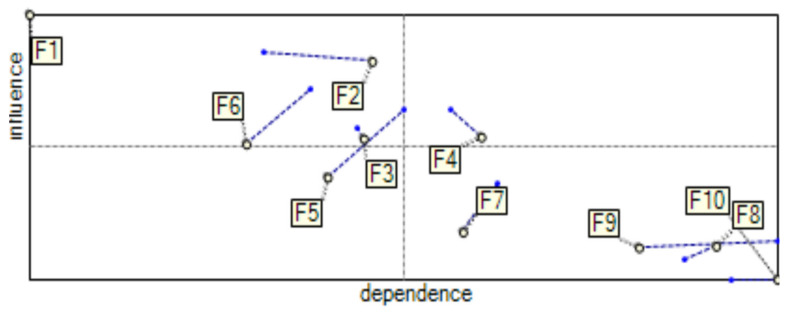
Displacement map (indirect to direct) for SCFs.

In Figure 7, the graphical displacement for analyzed SCFs is presented.

To conduct further analysis, a team of experts selected factors from the third group (linkage factors) and fourth group (driving factors): F_1_, F_2_, F_3_, F_4_ and F_6_ (F_1_—Management commitment; F_2_—Communication; F_3_—Rewards and Recognition; F_4_—Trust between managers and employees; F_6_—Education on OHS) (Figure 7) as key SCFs supporting the development of sustainability. These factors were used as the input data in the F-TOPSIS analysis.

### 4.3. Ranking of the Most Important Safety Culture Factors (F—TOPSIS)

In this part of the conducted study, five safety culture factors (F_1_—Management commitment; F_2_—Communication; F_3_—Rewards and Recognition; F_4_—Trust between managers and employees; F_6_—Education on OHS), identified in Chapter 4.3 as more important, were ranked from a sustainability perspective. For sustainable improvement in performance through safety culture, company must rank the critical safety culture factors based upon their relative importance.

As the ISM-MICMAC approach used in Chapter 4.3 did not take into account the relative importance of the factors to be classified, F-TOPSIS was used in this part of the study to rank them.

The first step of the study involved the selection of criteria for safety culture factor evaluation from a sustainability perspective. From a sustainability perspective, many factors can be taken into account when assessing the safety culture factors. However, sustainability cannot be assured without setting the context of the organization. As such, each company, based on its own context, should chose criteria for safety culture factor ranking.

On the basis of the context of the organization, and the goals set by the CEO, experts from the company identified the most important criteria from a sustainability perspective, according to which the assessment of safety culture factors was carried out (Table 14).

The decision makers (DM_1,_ DM_2_, DM_3_) used the linguistic variables (Table 4) to assess the importance of each criterion (range ‘‘very low” to ‘‘very high”) (Table 15).

The linguistic terms of each linguistic variable were converted into TFNs with the interval [0, 1] according to Table 16, and the aggregate fuzzy weights of each criterion given by three decision makers were calculated using Equation (10).

In the next step, the decision-makers from the company (DM_1_, DM_2_, DM_3_) used the linguistic rating variables (Table 5) to evaluate the rating of five safety culture factors: F_1_—Management commitment; F_2_—Communication; F_3_—Rewards and Recognition; F_4_—Trust between managers and employees; and F_6_—Education on OHS with respect to each criterion (shown in Table 14). Then, linguistic evaluation was transformed into TFN (shown in Table 5) to develop the fuzzy decision matrix. The results are presented in Table 17.

In the next stage of the conducted study, the fuzzy decision matrix (Table 18), normalized fuzzy decision matrix (Table 19) and weighted normalized matrix (Table 20) were computed.

After that, the distance of each alternative from the FPI matrix (d*) and FNI matrix (*d*^−^) was calculated using Equations (15)–(18), and the closeness coefficient ($CC_{i}$) for every alternative was calculated using Equation (19) (Table 21). Then, the factors were ranked according to the $CC_{i}$. The best alternative was closest to the FPIS and farthest from the FNIS.

**Figure 8:**
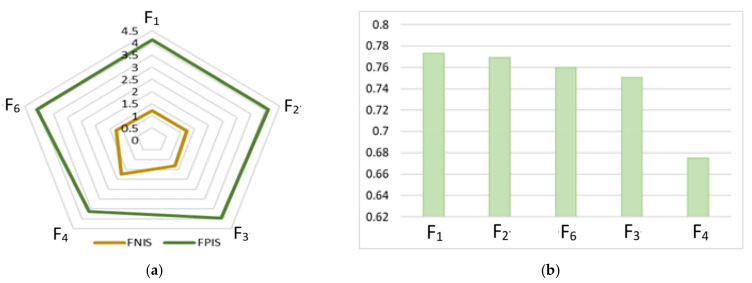
F-TOPSIS results: (**a**) FPIS and FNIS distance; (**b**) closeness ratio versus factor.

A comparison of $d_{1}^{*},\;d_{2}^{*},\;\ldots ,\;d_{5}^{*}$ and $d_{1}^{-},\;d_{2}^{-},\;\ldots ,\;d_{5}^{-}$ *d*-values for the analyzed culture safety factors is shown in Figure 8.

It is noted that factor F1 “Management commitment” has the highest closeness ratio, which means that is the highest ranked factor. Based on MICMAC approach, it was also identified as the most influential factor (Figure 6). The next most important identified factors are: F_2_, “Communication”, F_6_, “Education on OHS”, and F_3_, “Rewards and Recognition”. The smallest closeness ratio was identified for factor F_4_, “Trust between managers and employees”.

### 4.4. Sensitivity Analysis

The next step of the analyses was the sensitivity analysis. The sensitivity analysis was performed to assess the influence of the preferences given by the decision makers for the calculated closeness ratio. Sensitivity analysis for the criteria and alternatives was performed. During the analysis, 10 cases were conducted. In each case, the lowest rating was increased by one for each criterion/alternative (F_i_+/C_i_+)—for example, from V to VH—or the highest rating was decreased by one level for each criteria (F_i_−/C_i_−).

**Figure 9:**
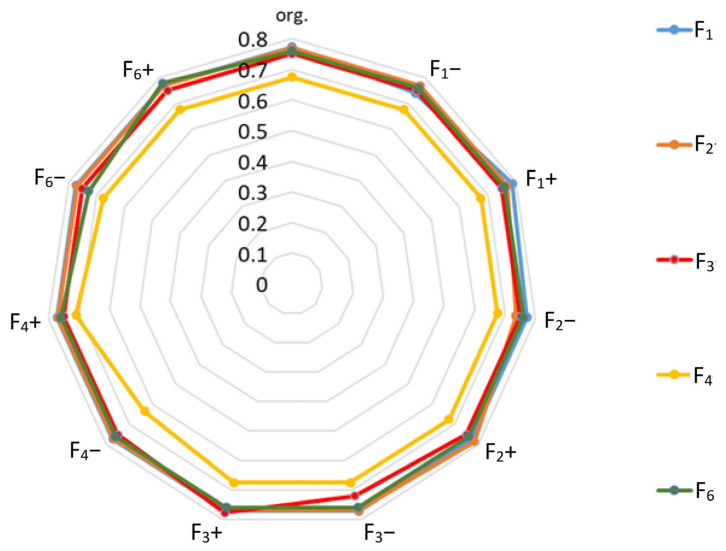
Sensitivity analysis results for the alternatives—closeness ratio versus factor.

The details of the obtained rankings of origin (without changes) results for the alternatives are shown in Table 22, and the closeness coefficient is shown in Figure 9.

When analyzing the obtained results, it should be noted that the dominant factors (F_1,_ F_2,_ F_3_, F_5_) changed their position in the ranking. This means that they should be treated as equally important due to slight differences in the closeness ratio. The visible difference in factors for the factor F_4_ did not result in its position in the ranking changing. This means that a slight underestimation or overestimation of the ratings by each of the decision-makers did not have a significant impact on the obtained ranking.

**Figure 10:**
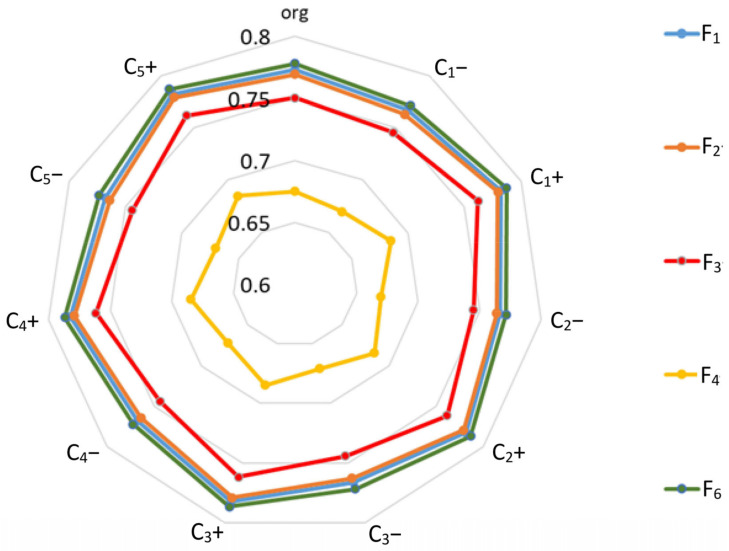
Sensitivity analysis results for the criteria—closeness ratio versus factor.

In Table 23, detailed rankings of origin (without changes) results for the criteria are presented. The closeness coefficient is shown in Figure 10.

The obtained results indicate that changes in the values of the criteria’s importance level do not affect the ranking of the factors. This confirms the preliminary hypothesis and means that the model is not sensitive to the adopted level of importance of the criteria. None of the analyzed factors (F_1,_ F_2,_ F_3_, F_5,_ F_6_) changed its position in the ranking.

## 5. Conclusions

The research question driving this study was “How can sustainability practices be improved by the development of safety culture?”, specifically “Which of the SCF have the greatest impact on sustainability improvement in the company?”.

According to [126], sustainability is one of the critical drivers for future development of business. As such, in recent years, many organizations have introduced or changed the rules of operation and modernized products and processes in order to meet the environmental and social challenges of sustainable development. However, many researchers indicate that the introduced changes are very often only superficial, and believe that in order to significantly contribute to the implementation of environmental and social challenges, enterprises will have to undergo cultural changes [127,128]. Therefore, they suggest adding another pillar, namely culture, to the three basic pillars of sustainable development (economic, environmental, social) [129,130]. An important part of the organizational culture is the safety culture; therefore, it plays an important role in strategic management [131,132], thus contributing to the achievement of its goals, including the goals of sustainable development.

In this study, an integrated approach (ISM, MICMAC and F-TOPSIS) was used to analyze the interactions between safety culture factors and to rank them from the perspective of the environmental, social and economic dimensions of sustainability. The objective of this study was to evaluate the safety culture factors and prioritize them, considering sustainability issues.

This approach was used in an automotive company to improve and develop the company’s practices aimed at implementing its sustainable development strategy. In real-world situations, many factors can influence the improvement of sustainability in an enterprise, and the strength of their impact will vary from organization to organization. As a consequence, decision-makers face the dilemma of what factors should be taken into account, what is the strength of their impact and how these factors are related to each other. In the surveyed company, based on a literature analysis and discussions with company experts, ten SCFs were identified to meet the challenges of sustainable development. Then, ISM-MICMAC analysis was employed to identify the key safety culture factors, based on their influence and dependence value. Among the analyzed factors, a team of experts identified the most important factors: F_1_, F_2_, F_6_, F_3_ and F_4_ (F_1_—Management commitment; F_2_—Communication; F_6_—Education on OHS; F_3_—Rewards and Recognition; F_4_—Trust between managers and employees;) as key SCFs supporting the development of sustainability. Finally, the results of ISM-MICMAC analysis were used as an input to rank the factors by means of the F-TOPSIS method.

The F-TOPSIS results indicate that “Management commitment” (F_1_) can be recognized as the most vital safety culture factor for improving sustainability performance in the company. It is identified that the proposed decision methodology has strategic importance in adopting sustainability practices in the industry.

From the perspective of management implications, the approach proposed in this publication can support decision-makers in many issues. Firstly, the proposed solution supports teamwork, because both the identification of factors influencing the safety culture and the identification of the relationships between them are carried out by a multidisciplinary team. Team discussion allows for a better understanding of both the safety culture factors and builds awareness of their importance in achieving sustainable company goals. Secondly, the ranking of solutions can provide a guide and support decision-makers in defining the policy of introducing measures to improve the safety culture and their integration with the environmental, social and economic goals of the company.

### Limitation and Future Research

The presented research of course has some limitations. Firstly, because the safety culture is multi-dimensional and multi-faceted concept and it depends on the country, geographical location and organizational culture of the company, the findings of the research are not universal. Secondly, the analysis was performed based on one company from the automotive industry and the experience of the chosen experts from this company, which represents an element of bias.

By reviewing the results of these studies in terms of theory and practice, directions for further research can be formulated. First, other methods such as fuzzy MICMAC, fuzzy VIKOR, fuzzy ANP or fuzzy ELETRE can be used in this type of research. Second, due to the intensive development of the Safety 4.0 concept and their possible impact on meeting sustainability challenges, in subsequent stages of research it would be necessary to consider the potential benefits of this concept in supporting SHE processes towards sustainability.

## Figures and Tables

**Table 3:** Safety culture factors/aspects.

Safety Culture Factors/Aspects	Author/s
(1) Top management commitment; (2) safety manager commitment; (3) worker commitment; (4) safety supervisor competence; (5) worker knowledge and experience; (6) worker empowerment; (7) safety communication; (8) safety incentive program; (9) incident reporting system; (10) housekeeping	[20]
(1) Management commitment; (2) communication; (3) rewards and recognition; (4) trust between managers and employees; (5) employee engagement; (6) education on OHS; (7) employees’ competences; (8) attitudes towards OHS regulations; (9) analysis of accidents; (10) monitoring employees’ behavior	[21]
(1) Psychological: safety attitude, peer influence, safety knowledge, perception of risk; (2) situational: safety rules, accident and incident, reporting, working environment, job satisfaction; (3) behavioral: management commitment, safety commitment, ownership of safety, safety training, safety communication, reward and recognition, safety investment, worker’s competencies	[61]
(1) Workplace safety perception; (2) safety implementation; (3) status of safety committee; (4) accountability of work; (5) worker involvement; (6) workers’ safety perception; (7) safety priority over other goals; (8) safety investigation; (9) safety policy; (10) rules and procedures; (11) risk assessment; (12) employee attitude toward safety; (13) safety communication; (14) safety training; (15) safety compliance and management commitment.	[62]
(1) Safety communication; (2) safety incentives; (3) safety manager’s attitude; (3) safety manager’s behavior; (4) safety compliance; (5) safety policy; (6) safety participation; (7) safety rules and procedures; (8) safety training; (9) safety worker’s involvement	[63]
(1) Safety management system and procedure; (2) management commitment; (3) safety attitudes; (4) workmate’s influences; (5) employee’s involvement; (6) safety knowledge; (7) safety behavior	[64]
(1) Training, briefing and competency; (2) vision, leadership and commitment; (3) law, rules and work procedures; (4) safety and crisis management; (5) individual agents; (6) management style and organizational communication; (7) participation and commitment of personnel, supervisors and middle management; (8) non-organizational agents; (9) making available foundations and source management.	[65]
(1) Management system: participation, safety mind, communication and information exchange, leadership, safety education, safety laws and regulations; (2) individual factors: knowledge, motivation, attitude, lifestyle, competence, responsibility, expert knowledge and skill; (3) organizational factors: production speed and timing, equipment, facilities, and technology, sources.	[66]

**Table 4:** Fuzzy linguistic terms and correspondent fuzzy numbers for each criterion.

Linguistic Terms	Abbreviation	TFN
Very low important	VL	(0, 0, 0.1)
Low important	L	(0, 0.1, 0.3)
Medium low	ML	(0.1, 0.3, 0.5)
Medium Important	M	(0.3, 0.5, 0.7)
Medium high	MH	(0.5, 0.7, 0.9)
High important	H	(0.7, 0.9, 1.0)
Very high important	VH	(0.9, 1.0, 1.0)

**Table 5:** Linguistic terms for alternative ratings.

Linguistic Terms	Abbreviation	TFN
Very poor	VP	(0, 0, 1)
Poor	P	(0, 1, 3)
Medium poor	MP	(1, 3, 5)
Fair	F	(3, 5, 7)
Medium good	MG	(5, 7, 9)
Good	G	(7, 9, 10)
Very good	VG	(9, 10, 10)

**Table 6:** Selected environmental and social risk associated with the automotive industry.

Sustainability Issues	Automotive				
	Metal Products and Machinery		Related Sectors		
			Iron, Steel and Other Metals	Electronic Products	Precision Instruments
Energy	M		H	M	M
Water use	M		M	M	M
Emission to water	H		M	H	H
Waste	H		H	M	M
Emission to Air	M		H	M	M
Ecosystems	L		L	L	L
Workplace health and safety	M		H	L	L
Disaster risk	M		M	L	L
Scoring rating:	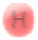	High risk issue	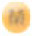 Medium risk issue 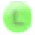		Low risk issue

Source: own elaboration based on [85].

**Table 7:** The factors affecting safety culture.

No.	Name of the Factor	Description	References
F_1_	Management commitment	This commitment can be manifested in the positive attitudes toward the activities relating to safety management and in the behaviours visible to the workers	[14,20,21,43,51,52,61,64,95,96,97,98,99,100,101]
F_2_	Communication	Transfer of information to employees about the possible risks in the workplace and the correct way to combat them.	[10,20,21,61,62,63,96,102,103,104]
F_3_	Rewards and Recognition	Fair incentive and feedback system that encourages employees to work safely	[20,21,38,51,61,97,104,105,106]
F_4_	Trust between managers and employees	Managers treat employees with respect, keep their promises and encourage mutual trust and support. Employees respect the orders of their superiors and follow their recommendations	[21,51,107,108]
F_5_	Employee engagement	Orientation towards active participation in safety. The workforce is engaged when individuals promote safe behaviors and actively reduce workplace hazards	[52,63,96,104,109,110,111,112]
F_6_	Education on OHS	Health and safety training, which are adapted to the specifics of the work (on-the-job training) and hazards occurring at the workplace. In addition, promoting safe behaviour through educational campaigns, posters and practical exercises	[20,52,63,65,66,95,97,113,114,115,116]
F_7_	Employees’ competences	Competence refers to the knowledge, skills and attitudes that are essential to the successful completion of a task.	[20,21,51,61,65,66,96,106,117,118]
F_8_	Attitudes towards OHS regulations	A sense of personal responsibility for the safety of each employee, attitude to health and safety regulations and compliance with them.	[14,20,21,62,63,66,95,112,115]
F_9_	Reporting and analysis of accidents	Reporting accidents and near misses and their analysis, taking into account the causes and effects of accidents, initiating corrective and preventive actions.	[20,21,38,61,97,99,113,119]
F_10_	Monitoring employees’ behavior	Monitoring the state of safety and supervision over the way work is performed (e.g., the use of protection measures and detection of potential problems).	[21,64,95,97,102,113,120,121,122,123]

**Table 8:** Factors comparison matrix (SSIM).

	F_2_	F_3_	F_4_	F_5_	F_6_	F_7_	F_8_	F_9_	F_10_
**F_1_**	V	V	V	V	V	V	V	V	V
**F_2_**	-	V	X	X	X	V	A	A	X
**F_3_**	-	-	V	O	X	V	X	O	O
**F_4_**	-	-	-	V	V	X	X	X	O
**F_5_**	-	-	-	-	O	X	O	O	V
**F_6_**	-	-	-	-	-	V	V	V	V
**F_7_**	-	-	-	-	-	-	V	O	V
**F_8_**	-	-	-	-	-	-	-	O	O
**F_9_**	-	-	-	-	-	-	-	-	V

**Table 10:** Final reachability matrix.

Factors	F_1_	F_2_	F_3_	F_4_	F_5_	F_6_	F_7_	F_8_	F_9_	F_10_	Driving Power
**F_1_**	1	1	1	1	1	1	1	1	1	1	10
**F_2_**	0	1	1	1	1	1	1	1 *	1 *	1	9
**F_3_**	0	0	1	1	0	1	1	1	1 *	0	6
**F_4_**	0	1	0	1	1	1 *	1	1	1	1 *	8
**F_5_**	0	1	0	0	1	0	1	1 *	1 *	1 *	6
**F_6_**	0	1	1	0	0	1	1	1	1	1	7
**F_7_**	0	0	0	1	1	0	1	1	0	1	5
**F_8_**	0	0	1	1	0	0	0	1	0	0	3
**F_9_**	0	1	0	1	0	0	0	0	1	1	4
**F_10_**	0	1	0	0	0	0	0	1	0	1	3
**Dependence**	1	7	5	7	5	5	7	9	7	8	61

* means value after applying transitivity.

**Table 11:** Level partition of the SCFs.

Factors	R(t_i_)—Reachability Set	A(t_i_)—Antecedent Set	R(t_i_) ∩ A(t_i_)	Level
**F_1_**	1, 2, 3, 4, 5, 6, 7, 8, 9, 10	1	1	VII
**F_2_**	2, 3, 4, 5, 6, 7, 8, 9, 10	2, 4, 5, 6, 9, 10	2, 4, 5, 6, 9, 10	VII
**F_3_**	2, 3, 4, 5, 6, 7, 8, 9	1, 2, 3, 6, 8	3, 6, 8	VI
**F_4_**	2, 4, 5, 6, 7, 8, 9, 10	1, 2, 3, 4, 7, 8, 9	2, 4, 7, 8, 9	V
**F_5_**	2, 5, 6, 7, 8, 9, 10	1, 2, 3, 4, 5, 7	2, 5, 7	IV
**F_6_**	2, 3, 6, 7, 8, 9, 10	1, 2, 3, 4, 5, 6	2, 3, 6	III
**F_7_**	4, 5, 7, 8, 10	1, 2, 3, 4, 5, 6, 7	4, 5, 7	II
**F_8_**	3, 4, 8	1, 2, 3, 4, 5, 6, 7, 8	3, 4, 8	I
**F_9_**	2, 4, 9, 10	1, 2, 3, 4, 5, 6, 9	2, 4, 9	II
**F_10_**	2, 10	1, 2, 4, 5, 6, 7, 9, 10	2, 10	I

**Table 12:** The strength of the influence of SCF.

	F_1_	F_2_	F_3_	F_4_	F_5_	F_6_	F_7_	F_8_	F_9_	F_10_
**F_1_**	0	0	2	2	3	2	1	1	3	2
**F_2_**	0	0	2	1	2	2	1	2	3	1
**F_3_**	0	0	0	1	0	1	2	1	2	0
**F_4_**	0	1	0	0	2	1	2	1	2	2
**F_5_**	0	1	0	0	0	0	2	2	3	3
**F_6_**	0	1	1	0	0	0	2	3	3	2
**F_7_**	0	0	0	1	1	0	0	2	0	3
**F_8_**	1	1	2	1	0	0	0	0	0	0
**F_9_**	0	1	0	1	0	0	0	0	0	2
**F_10_**	0	1	0	0	0	0	0	1	0	0

**Table 13:** The matrix of indirect influence of SCFs.

	F_1_	F_2_	F_3_	F_4_	F_5_	F_6_	F_7_	F_8_	F_9_	F_10_
**F_1_**	0	2947	2959	4051	2603	1876	3770	6007	5319	6727
**F_2_**	0	2544	2530	3451	2205	1586	3190	5144	4515	5748
**F_3_**	0	1805	1761	2433	1570	1176	2364	3660	3301	3914
**F_4_**	0	1843	1772	2457	1593	1135	2359	3717	3296	4171
**F_5_**	0	1508	1407	1934	1278	911	1831	2931	2546	3215
**F_6_**	0	1829	1736	2350	1533	1109	2212	3570	3105	3872
**F_7_**	0	948	921	1246	841	591	1163	1898	1647	2121
**F_8_**	0	787	835	1078	707	530	1003	1595	1433	1705
**F_9_**	0	783	771	1022	716	524	1032	1584	1444	1695
**F_10_**	0	507	478	624	422	319	650	1016	922	1044

**Table 14:** The criteria for safety culture factors evaluation.

No	Criteria
C_1_	Decries risk in the workplace
C_2_	Safety consciousness
C_3_	Pro-environmental—behavior
C_4_	Waste reduction
C_5_	Productivity

**Table 15:** Each criterion weight in linguistic term.

DM	Criteria				
	C_1_	C_2_	C_3_	C_4_	C_5_
DM_1_	VH	VH	VH	MH	M
DM_2_	H	VH	VH	VH	M
DM_3_	VH	VH	VH	H	VH

**Table 16:** Each criterion’s weight in TFN.

DM	Criteria				
	C_1_	C_2_	C_3_	C_4_	C_5_
DM_1_	(0.9, 1.0, 1.0)	(0.9, 1.0, 1.0)	(0.9, 1.0, 1.0)	(0.5, 0.7, 0.9)	(0.3, 0.5, 0.7)
DM_2_	(0.7, 0.9, 1.0)	(0.9, 1.0, 1.0)	(0.9, 1.0, 1.0)	(0.9, 1.0, 1.0)	(0.3, 0.5, 0.7)
DM_3_	(0.9, 1.0, 1.0)	(0.9, 1.0, 1.0)	(0.9, 1.0, 1.0)	(0.7, 0.9, 1.0)	(0.9, 1.0, 1.0)
Weight	(0.83, 0.97, 1)	(0.9, 1, 1)	(0.9, 1, 1)	(0.7, 0.87, 0.97)	(0.5, 0.67, 0.8)

**Table 17:** The linguistic ratings of the six factors by DM under all criteria.

DM	Factor	C_1_	C_2_	C_3_	C_4_	C_5_	Factor	C_1_	C_2_	C_3_	C_4_	C_5_
DM_1_	F_1_	G	VG	VG	G	G	F_1_	(7, 9, 10)	(9, 10, 10)	(9, 10, 10)	(7, 9, 10)	(7, 9, 10)
	F_2_	VG	G	VG	G	G	F_3_	(9, 10, 10)	(7, 9, 10)	(9, 10, 10)	(7, 9, 10)	(7, 9, 10)
	F_3_	F	F	G	VG	VG	F_2_	(3, 5, 7)	(3, 5, 7)	(7, 9, 10)	(9, 10, 10)	(9, 10, 10)
	F_4_	G	G	F	F	G	F_4_	(7, 9, 10)	(7, 9, 10)	(3, 5, 7)	(3, 5, 7)	(7, 9, 10)
	F_6_	VG	VG	G	G	MG	F_6_	(9, 10, 10)	(9, 10, 10)	(7, 9, 10)	(7, 9, 10)	(5, 7, 9)
DM_2_	F_1_	G	VG	VG	G	G	F_1_	(7, 9, 10)	(9, 10, 10)	(9, 10, 10)	(7, 9, 10)	(7, 9, 10)
	F_2_	VG	G	VG	G	G	F_3_	(9, 10, 10)	(7, 9, 10)	(9, 10, 10)	(7, 9, 10)	(7, 9, 10)
	F_3_	G	VG	VG	VG	VG	F_2_	(7, 9, 10)	(9, 10, 10)	(9, 10, 10)	(9, 10, 10)	(9, 10, 10)
	F_4_	G	G	G	F	G	F_4_	(7, 9, 10)	(7, 9, 10)	(7, 9, 10)	(3, 5, 7)	(7, 9, 10)
	F_6_	VG	VG	G	G	MG	F_6_	(9, 10, 10)	(9, 10, 10)	(7, 9, 10)	(7, 9, 10)	(5, 7, 9)
DM_3_	F_1_	VG	VG	VG	G	G	F_1_	(9, 10, 10)	(9, 10, 10)	(9, 10, 10)	(7, 9, 10)	(7, 9, 10)
	F_2_	VG	G	VG	G	VG	F_3_	(9, 10, 10)	(7, 9, 10)	(9, 10, 10)	(7, 9, 10)	(9, 10, 10)
	F_3_	VG	VG	VG	VG	VG	F_2_	(9, 10, 10)	(9, 10, 10)	(9, 10, 10)	(9, 10, 10)	(9, 10, 10)
	F_4_	G	G	G	G	G	F_4_	(7, 9, 10)	(7, 9, 10)	(7, 9, 10)	(7, 9, 10)	(7, 9, 10)
	F_6_	VG	VG	VG	G	G	F_6_	(9, 10, 10)	(9, 10, 10)	(9, 10, 10)	(7, 9, 10)	(7, 9, 10)

**Table 18:** The fuzzy decision matrix and fuzzy weights of five factors.

Factor	C_1_	C_2_	C_3_	C_4_	C_5_
F_1_	(7.67, 9.33, 10)	(9, 10, 10)	(9, 10, 10)	(7, 9, 10)	(7, 9, 10)
F_2_	(9, 10, 10)	(7, 9, 10)	(9, 10, 10)	(7, 9, 10)	(7.67, 9.33, 10)
F_3_	(6.33, 8, 9)	(7, 8.33, 9)	(8.33, 9.67, 10)	(9, 10, 10)	(9, 10, 10)
F_4_	(7, 9, 10)	(7, 9, 10)	(5.67, 7.67, 9)	(4.33, 6.33, 8)	(7, 9, 10)
F_6_	(9, 10, 10)	(9, 10, 10)	(7.67, 9.33, 10)	(7, 9, 10)	(5.67, 7.67, 9.33)
weight	(0.83, 0.97, 1)	(0.9, 1, 1)	(0.9, 1, 1)	(0.7, 0.87, 0.97)	(0.5, 0.67, 0.8)

**Table 19:** The normalized fuzzy decision matrix.

Factor	C_1_	C_2_	C_3_	C_4_	C_5_
F_1_	(0.77, 0.93, 1)	(0.9, 1, 1)	(0.9, 1, 1)	(0.7, 0.9, 1)	(0.7, 0.9, 1)
F_2_	(0.9, 1, 1)	(0.7, 0.9, 1)	(0.9, 1, 1)	(0.7, 0.9, 1)	(0.77, 0.93, 1)
F_3_	(0.63, 0.8, 0.9)	(0.7, 0.83, 0.9)	(0.83, 0.97, 1)	(0.9, 1, 1)	(0.9, 1, 1)
F_4_	(0.7, 0.9, 1)	(0.7, 0.9, 1)	(0.57, 0.77, 0.9)	(0.43, 0.63, 0.8)	(0.7, 0.9, 1)
F_6_	(0.9, 1, 1)	(0.9, 1, 1)	(0.77, 0.93, 1)	(0.7, 0.9, 1)	(0.57, 0.77, 0.93)

**Table 20:** The fuzzy weighted normalized decision matrix.

Factor	C_1_	C_2_	C_3_	C_4_	C_5_
F_1_	(0.64, 0.9, 1)	(0.81, 1, 1)	(0.81, 1, 1)	(0.49, 0.78, 0.97)	(0.35, 0.6, 0.8)
F_2_	(0.75, 0.97, 1)	(0.63, 0.9, 1)	(0.81, 1, 1)	(0.49, 0.78, 0.97)	(0.38, 0.62, 0.8)
F_3_	(0.53, 0.77, 0.9)	(0.63, 0.83, 0.9)	(0.75, 0.97, 1)	(0.63, 0.87, 0.97)	(0.45, 0.67, 0.8)
F_4_	(0.58, 0.87, 1)	(0.63, 0.9, 1)	(0.51, 0.77, 0.9)	(0.3, 0.55, 0.77)	(0.35, 0.6, 0.8)
F_6_	(0.75, 0.97, 1)	(0.81, 1, 1)	(0.69, 0.93, 1)	(0.49, 0.78, 0.97)	(0.28, 0.51, 0.75)

**Table 21:** The $CC_{i}$ of the five factors.

	F_1_	F_2_	F_3_	F_4_	F_6_
$d_{i}^{*}$	1.21216	1.23105	1.31153	1.74415	1.28143
$d_{i}^{-}$	4.12514	4.10746	3.94634	3.62371	4.05613
$CC_{i}$	0.77289	0.76940	0.75056	0.67508	0.75992
Ranking	1	2	4	5	3

**Table 22:** Sensitivity analysis results for the alternatives—the factors’ ranking.

Rank	Original Rank	F_1_−	F_1_+	F_2_−	F_2_+	F_3_−	F_3_+	F_4_−	F_4_+	F_5_−	F_5_+
1	F_1_	F_2_	F_1_	F_1_	F_2_	F_1_	F_3_	F_1_	F_1_	F_1_	F_6_
2	F_2_	F_6_	F_2_	F_6_	F_1_	F_2_	F_1_	F_2_	F_2_	F_2_	F_1_
3	F_6_	F_3_	F_6_	F_3_	F_6_	F_6_	F_2_	F_6_	F_6_	F_3_	F_2_
4	F_3_	F_1_	F_3_	F_2_	F_3_	F_3_	F_6_	F_3_	F_3_	F_6_	F_3_
5	F_4_	F_4_	F_4_	F_4_	F_4_	F_4_	F_4_	F_4_	F_4_	F_4_	F_4_

**Table 23:** Sensitivity analysis results for the criteria—the factors’ ranking.

Rank	Original Rank	C1−	C1+	C2−	C2+	C3−	C3+	C4−	C4+	C5−	C5+
1	F_6_	F_6_	F_6_	F_6_	F_6_	F_6_	F_6_	F_6_	F_6_	F_6_	F_6_
2	F_1_	F_1_	F_1_	F_1_	F_1_	F_1_	F_1_	F_1_	F_1_	F_1_	F_1_
3	F_2_	F_2_	F_2_	F_2_	F_2_	F_2_	F_2_	F_2_	F_2_	F_2_	F_2_
4	F_3_	F_3_	F_3_	F_3_	F_3_	F_3_	F_3_	F_3_	F_3_	F_3_	F_3_
5	F_4_	F_4_	F_4_	F_4_	F_4_	F_4_	F_4_	F_4_	F_4_	F_4_	F_4_

## Data Availability

Not applicable.
